# Tracking the foreign body, a rare cause of hepatic abscess

**DOI:** 10.1186/1471-230X-14-167

**Published:** 2014-09-27

**Authors:** Pierre-François Laterre, Carole Dangoisse

**Affiliations:** Department of Critical Care Medicine, Cliniques Universitaires Saint Luc, Université Catholique de Louvain (UCL), Avenue Hippocrate, 10, 1200 Brussels, Belgium; St Thomas’ Hospital, Westminster Bridge Road, SE1 7EH London, UK

**Keywords:** Foreign body, Fishbone, Liver abscess, Perforation

## Abstract

**Background:**

Foreign body ingestion complicated by perforation of the digestive tract is a well-known occurrence. Contrary to this, perforation by fishbones has most often been described in South East Asian populations, and has the unusual characteristic of often being paucisymptomatic until secondary complications occur.

**Case presentation:**

We report the case of a 56 year-old man of asian origin who presented with a liver abscess of unknown origin, complicated by septic shock with multiorgan failure. He was later found to have a fishbone impacted in the left lobe of the liver, which had perforated the stomach and gone by unnoticed until presentation. The fishbone was extracted through laparotomy and the abscess was drained.

**Conclusion:**

This report highlights a cause of liver abscesses which is likely underreported in Northern European populations and which, although rare in occurrence, should be part of our diagnostic algorithm of “cryptogenic abscesses” since surgical removal of the foreign object as drainage warrant definitive treatment.

## Background

Ingestion of foreign bodies is a common problem but unintentional ingestion of fish bones forms a distinctive subcategory is more frequently encountered in Asian and Pacific populations, where consumption of fish largely surpasses that of Northern European populations. Approximately 80-90% of ingested foreign bodies pass through the gastrointestinal tract unrecognized, generally within one week [1]. Obstruction or perforation by foreign bodies is thought to occur in less than 1% of cases [2]. The main risk factor for their ingestion is the use of dentures impairing the sensory feedback of the palate. Other conditions include eating quickly, very young and old age, alcohol intoxication, hot or cold beverages, cognitive impairment and severe psychiatric conditions [3, 4]. The shape or size of objects cannot predict the risk of perforation as opposed to spontaneous passage [5]. Blunt objects can perforate the mucosa after erosion due to pressure from longstanding impaction [6]. The majority of fishbones that do become impacted lodge themselves in the upper aerodigestive tract present acutely and are referred to oto-rhino-laryngologists [7]. Below the gastro-oesophageal junction, fishbones tend to impact themselves in acute angles that slow their progression. Other predisposing anatomical factors for impaction are diverticulum-like cavities, hernias or neoplastic obstructions [3].

Liver abscesses caused by ingestion and migration of a foreign object are rare; although likely underreported, only 60 cases had been published by 2010. Less than half of these (44%) had been caused by fishbones [4]. Most liver abscesses are caused by direct penetration of the foreign object through the gastrointestinal tract, the stomach being the most common site [1], but they can also be a secondary focus of infection through bacteraemia from a distant site of perforation [8]. The force of peristaltic waves in the stomach likely propels fishbones into and then through the gastric wall, preferably at the antrum [9]. Due to its anatomical proximity with the stomach, the left lobe of the liver is the most frequent site where fishbones get impacted [1]. The thicker wall of the stomach allows the fishbone to penetrate the mucosa gradually while the omentum and neighbouring organs progressively surround and therefore seal the perforation [3]. Such a slow progression can account for the fact that the perforation in itself is often paucisymptomatic and therefore undiagnosed. In more than half of cases, there is a delay of more than two weeks between fishbone ingestion and symptomatic presentation [10].

Clinical presentation is non specific. In a recent review of the literature, only 12% of the patients had a suggestive medical anamnesis [4]. Patients present with vague abdominal symptoms such as epigastric pain, fever and chills, anorexia, nausea and vomiting, or even weight loss and fatigue [1]. They may also have had blood-streaked or coffee-ground emesis or be jaundiced [4]. Routine laboratory tests are equally non specific. The common diagnosis before surgery is often an acute appendicitis or diverticulitis [3].

CT-scan is the gold standard for diagnosis, fishbones appearing as calcified linear structures [11]. Yet they can easily be missed due to their small size and overlapping of tissues and/or fluids, even more so if the radiologist or the clinician is unaware of the potential diagnosis, such as in our case report. Other sources of error are the fishbone being obscured by oral contrast, and the resemblance of tiny bones to blood vessels. To bypass such causes of error, it is advised to perform thinner reconstructions (3 mm/1.5 mm) and coronal or sagittal views, as well as unenhanced images [7]. Perforations are often sealed and therefore the absence of indirect signs such as free air under the diaphragm can be falsely reassuring [3].

Despite the sensitivity of CT, pre-operative diagnosis remains poor in recent reviews, averaging 25% [3]. When a liver abscess is diagnosed, the presence of a foreign object as the source of the infection should be suspected if, in the previous 3 months, the patient has complained of epigastralgias of unknown origin or has had hematemesis, or if the patient shows more than one of the following characteristics: left lobe liver abscess, single location, no comorbidities, failure of antibiotic-based treatment, indirect signs of foreign object perforation on CT (thickened gastrointestinal walls, fistulous tract), or adherences between the liver and the gastrointestinal tract during surgery [4].

Treatment consists of culture-guided antibiotherapy, drainage, abcess and laparotomy for fishbone removal followed if necessary by the perforation site repair. Germs responsible for abscess formation are typically found in the normal flora of the human oral cavity, or alternatively those of the lower gut flora [1]. Wide-spectrum antibiotics should be initiated on presentation; then guided by culture of aspirates. A conservative approach consisting of radiological-guided percutaneous aspiration and drainage of the abscess can be considered [12, 13], but if the fishbone is not extracted, it must be regarded as an ongoing risk for organ injury and microbial colonisation [1].

The mortality in a recent review of 17 cases of liver abscesses secondary to fishbone perforation was 17,6% [1]. This high mortality rate underscores the difficulty of diagnosis and the need for awareness of this rare occurrence.

## Case presentation

We report the case of a 56-year-old man of asian origin presenting to ED with dyspnea and high fever. He had a history of psoriasis treated with low-dose corticosteroids (Methylprednisolone 4 mg every other day). Three days before admission, he started having chills and became progressively unwell. Vitals upon admission: blood pressure 70/30 mmHg, pulse 145 bpm, temperature 39,5°C, respiratory rate 28 per minute, O2 saturation 95%. The abdomen was soft to palpation yet mildly tender in the right upper quadrant. There was no sign of peritonitis. Laboratory investigations showed marked inflammation (C-reactive protein 32 mg/dl), leucocytosis (20 200 WBC/μL, 18 300 neutrophils/μL), thrombocytopenia (platelets 52 000/μL), coagulation disorder (INR 1.5) and elevated liver enzymes (aspartate aminotransferase 538 IU/mL, alanine aminotransferase 384 IU/mL, LDH 1138 IU/mL). Plain radiograph of the chest revealed right inferior lobe shadowing suggesting a possible pneumonia.

**Figure 1:**
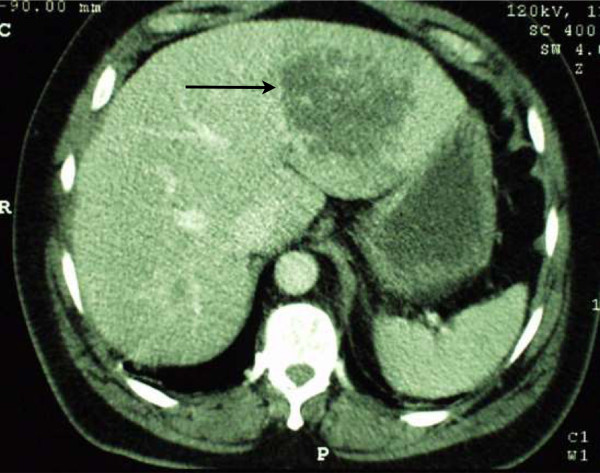
**Liver abscess: aspect on admission to hospital.** Transverse abdominal CT image of the liver revealing a hypodense area in the left lobe of the liver, measuring 85 mm in width, representing a solid liver abscess.

After ICU admission, a contrast enhanced CT-scan of the abdomen was performed since it revealed a left-sided solid liver abscess (Figure 1). There was no free liquid in the abdominal cavity, and no signs of pneumoperitoneum or perforation. The diagnosis at that point was of septic shock secondary to a liver abscess of unclear aetiology, complicated by acute lung injury and disseminated intravascular coagulation. The patient was started on an antibiotherapy course (Amoxicillin-Clavulanic acid), low-dose steroids and recombinant activated protein C, and improved during the first 48 hours.

**Figure 2:**
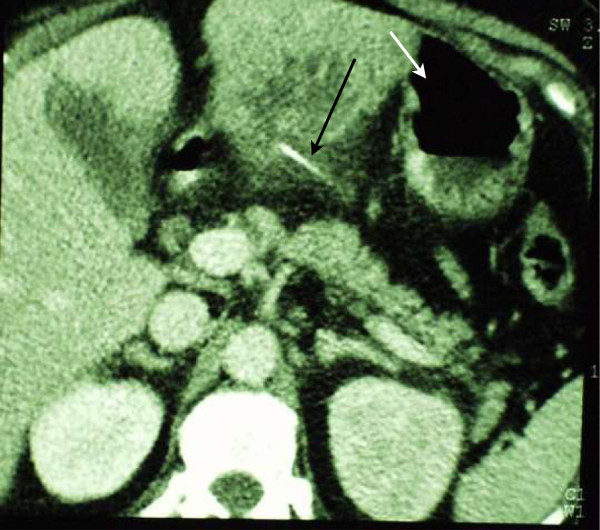
**Fishbone in the centre of the abscess.** Transverse abdominal CT image of the left lobe of the liver revealing the presence of a fishbone (calcified linear density, black arrow) in the centre of the abscess. Note the proximity of the stomach to the abscess (white arrow).

On day 3 the patient’s condition deteriorated. Abdominal CT scan was repeated. This time, the attention was drawn to a calcified density at the centre of the liver abscess not previously noticed (Figure 2). The structure was linear, measured 27 mm in length, and was in the left lobe of the liver protruding outwards, close to the duodenum.

**Figure 3:**
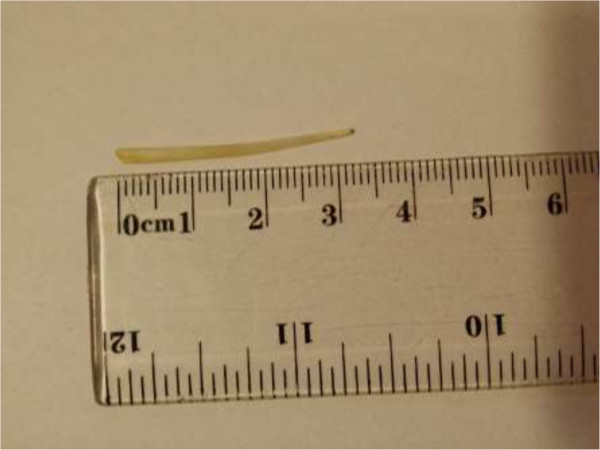
**Fishbone extracted by laparotomy.** The fishbone that was removed from the liver measured 3 cm in length.

An upper gastro-intestinal endoscopy showed no perforation, on foreign body. An exploratory laparoscopy was carried out. The left lower lobe of the liver was found to adhere closely to the anterior aspect of the lesser curvature of the stomach. A small quantity of pus was extracted from under the adherences, but the foreign body could not be reached by laparoscopy. The surgery was therefore converted to a laparotomy and a calcified foreign object measuring 3 cm in length removed from in the liver (Figure 3). This object was subsequently identified as being a fish bone. Upon careful retro anamnesis, the patient remembered having felt severe epigastric pain during a meal eaten seven days prior to admission, pain which had rapidly resolved without any specific treatment.

Drainage tubes were placed in the abscess for postoperative drainage. Blood cultures taken on admission grew Streptococcus hemolyticus (group G) and Streptococcus sanguis. The antibiotherapy was therefore shifted to Penicillin. The patient was discharged from the Intensive Care Unit 11 days after admission.

On day 13, he became pyrexial again. Imaging showed a slight enlargement of the collection with a heterogeneous liquid component, occupying the entire left lobe of the liver. A percutaneous drain was inserted under ultrasound-guided control. Culture of material grew Staphylococcus coagulase negative. Intravenous antibiotherapy with Penicillin was administered during the entire stay, and shifted to Amoxicillin-Clavulanic acid for another ten days upon discharge from the hospital, on day 43. Subsequent controls of the CT-scan confirmed a slow regression of the collection. The patient was last seen six months after the episode; he had remained symptom-free.

## Conclusions

This case report illustrates an uncommon cause of liver abcess rarely observed in the westerns world. Fishbones ingestion responsible of this complications are more often described in the Asian Literature. This clinical case also emphasises the fact that, although we have access to powerful imagery methods, the diagnosis of foreign body ingestion still remains a confounding one. Indeed, many liver abscesses secondary to perforation are still being misdiagnosed as cryptogenic, thereby impending a correct management. Failure to recognise this particular condition has serious repercussions, as shows the high mortality due to perforation and/or septic shock.

## Consent

Written informed consent was obtained from the patient for publication of this Case report and any accompanying images. A copy of the written consent is available for review by the Editor of this journal.

## Abbreviations

bpm: Beats per minute
CT: Computed tomography
LDH: Lactate dehydrogenase
ICU: Intensive care unit
INR: International normalized ratio
SIRS: Systemic inflammatory response syndrome
WBC: White blood cells.
