# Erratum: Tracking the foreign body, a rare cause of hepatic abscess

**DOI:** 10.1186/s12876-015-0252-z

**Published:** 2015-02-26

**Authors:** Carole Dangoisse, Pierre-François Laterre

**Affiliations:** Department of Critical Care Medicine, Cliniques Universitaires Saint Luc, Université Catholique de Louvain (UCL), Avenue Hippocrate, 10, 1200 Brussels, Belgium

## Erratum

After publication of this Case Report [1], we noticed a mistake in the order of the authors. Carole Dangoisse was meant to be the first author on this article and Pierre-François Laterre the last author. Also there were mistakes in the affiliation and contact details for Carole Dangoisse. This has been now corrected.
